# Review on optofluidic microreactors for artificial photosynthesis

**DOI:** 10.3762/bjnano.9.5

**Published:** 2018-01-04

**Authors:** Xiaowen Huang, Jianchun Wang, Tenghao Li, Jianmei Wang, Min Xu, Weixing Yu, Abdel El Abed, Xuming Zhang

**Affiliations:** 1 Energy Research Institute, Shandong Academy of Sciences, Jinan, Shandong 250014, China; 2 Department of Applied Physics, The Hong Kong Polytechnic University, Hong Kong, China; 3 The Hong Kong Polytechnic University Shenzhen Research Institute, Shenzhen 518057, China; 4 Key Laboratory of Spectral Imaging Technology, Xi’an Institute of Optics and Precision Mechanics, Chinese Academy of Sciences, Xi’an, Shaanxi 710119, China,; 5 Laboratoire de Photonique Quantique et Moléculaire, UMR 8537, Ecole Normale Supérieure de Cachan, CentraleSupélec, CNRS, Université Paris-Saclay, 61 avenue du Président Wilson, 94235 Cachan, France

**Keywords:** artificial photosynthesis, carbon dioxide fixation, coenzyme regeneration, microfluidics, optofluidics, water splitting

## Abstract

Artificial photosynthesis (APS) mimics natural photosynthesis (NPS) to store solar energy in chemical compounds for applications such as water splitting, CO_2_ fixation and coenzyme regeneration. NPS is naturally an optofluidic system since the cells (typical size 10 to 100 µm) of green plants, algae, and cyanobacteria enable light capture, biochemical and enzymatic reactions and the related material transport in a microscale, aqueous environment. The long history of evolution has equipped NPS with the remarkable merits of a large surface-area-to-volume ratio, fast small molecule diffusion and precise control of mass transfer. APS is expected to share many of the same advantages of NPS and could even provide more functionality if optofluidic technology is introduced. Recently, many studies have reported on optofluidic APS systems, but there is still a lack of an in-depth review. This article will start with a brief introduction of the physical mechanisms and will then review recent progresses in water splitting, CO_2_ fixation and coenzyme regeneration in optofluidic APS systems, followed by discussions on pending problems for real applications.

## Review

### Introduction

**Figure 1:**
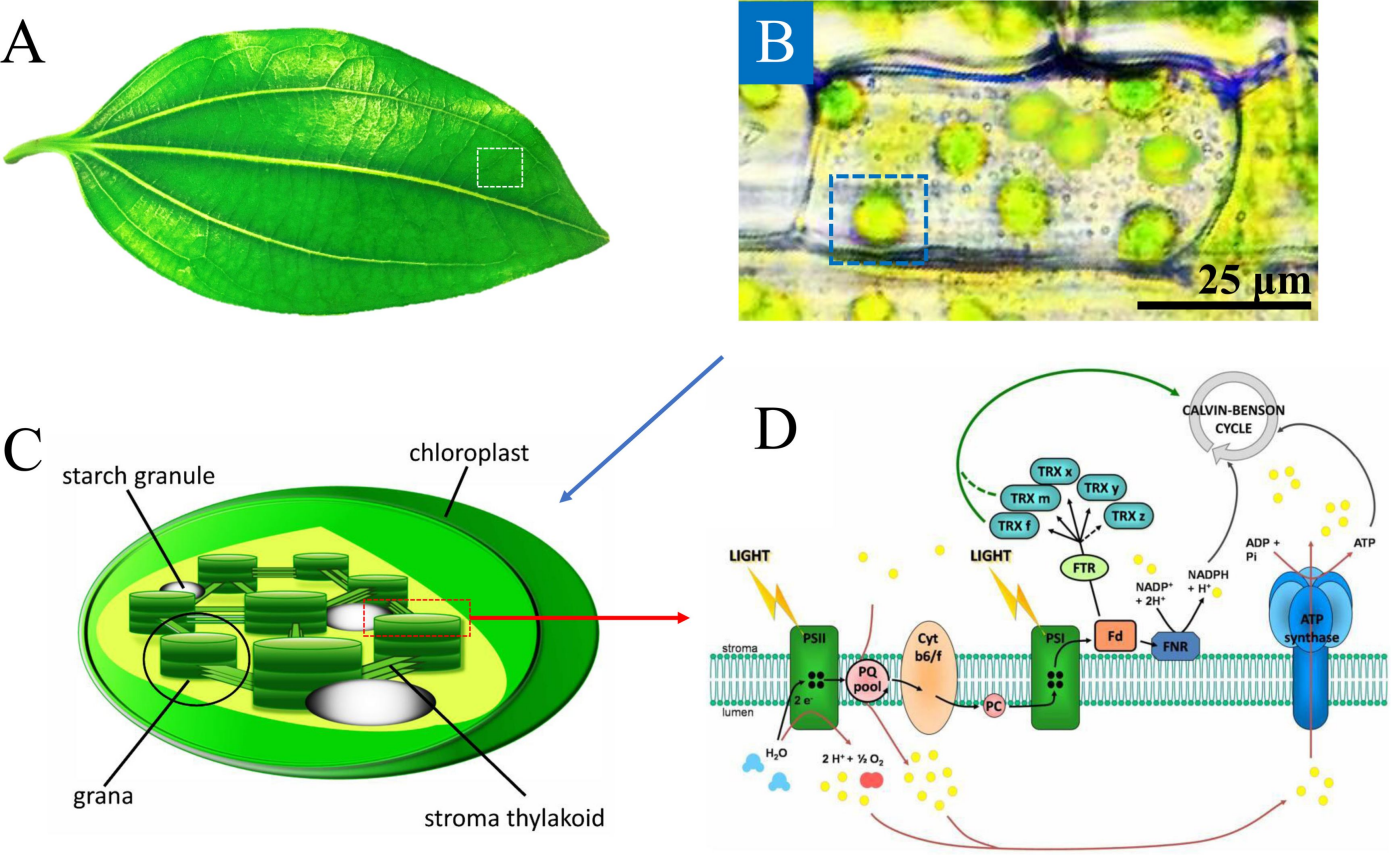
(A) A typical plant leaf. (B) Chloroplasts inside the plant cells. The average size of the chloroplasts is 6 µm (ranging from 3 to 10 µm). (C) Plant cell chloroplast structure. Adapted from [22], copyright BioMed Central Ltd. 2014. (D) Thylakoid membrane containing photosystem II reaction centers P680 and photosystem I reaction centers P700. Adapted from [23], copyright 2013 Michelet, Zaffagnini, Morisse, Sparla, Pérez-Pérez, Francia, Danon, Marchand, Fermani, Trost, and Lemaire.

The emerging energy crisis, the greenhouse effect and food shortage are devastating problems to be solved, and artificial photosynthesis (APS) is considered to be the most promising and viable method [1–9]. As the name implies, APS is the human replication of natural photosynthesis (NPS). NPS is a very important process in plants and other organisms which utilize sunlight, water and CO_2_ to synthesize energy-rich carbohydrates [10–11]. The chloroplast is the place where NPS occurs. To clearly introduce this organelle, progressively smaller structures (plant cell, chloroplast, thylakoid membrane) of a general leaf are shown in Figure 1A–D. Each chloroplast (Figure 1C) contains numerous thylakoids. On the thylakoid membrane, the natural light-harvesting antenna complexes, photosystem II (PS II, P680) and photosystem I (PS I, P700), capture the photons and regenerate the coenzyme for carbohydrates synthesis (Figure 1D). However, in APS, sunlight is used to create not only the carbohydrates but also other high-value chemicals from abundant resources [12–21].

**Figure 2:**
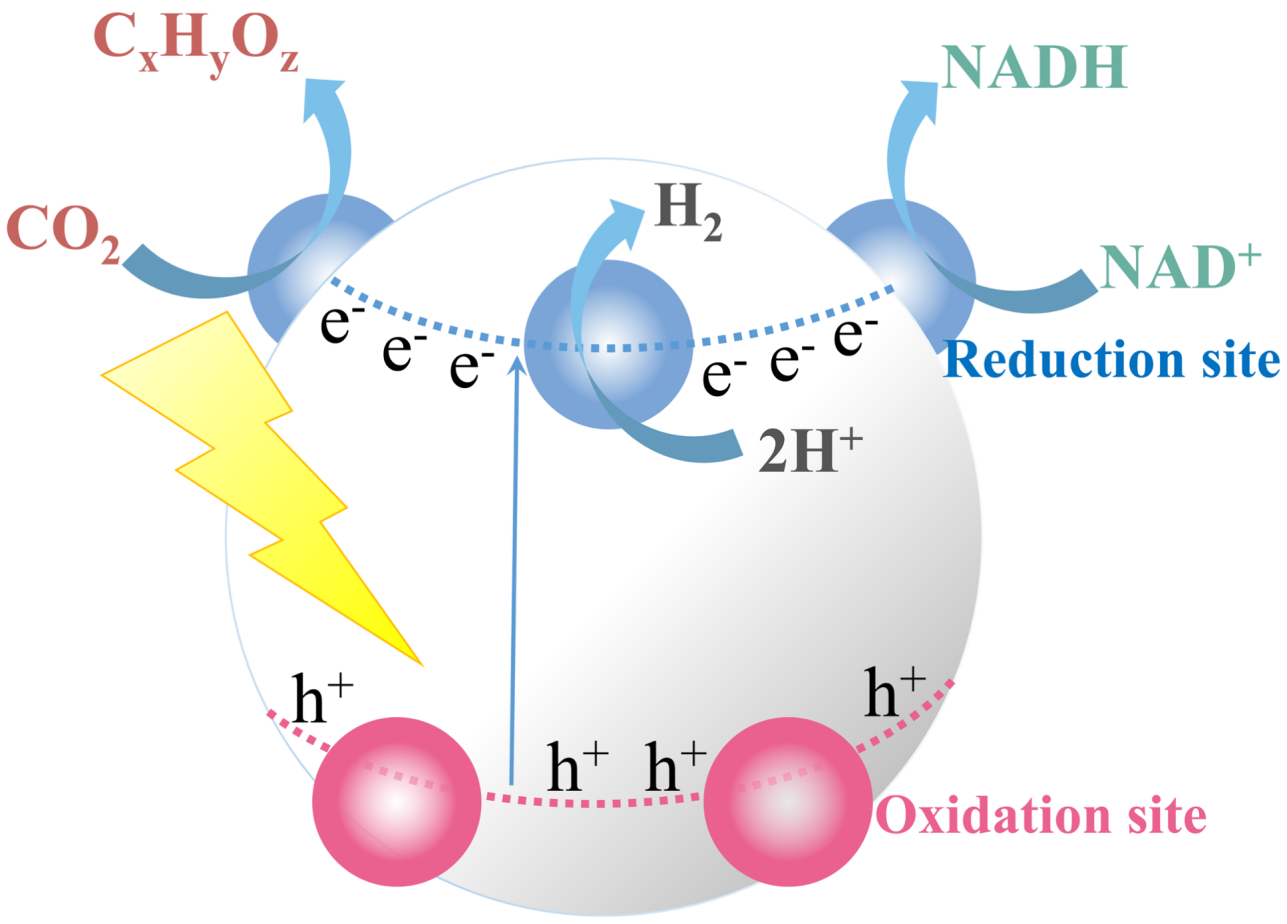
Basic principles and reactions in artificial photosynthesis, in which the processes of water splitting, CO_2_ reduction and coenzyme regeneration all utilize electrons on the reduction site.

Based on the targeted production, three key areas in the field of APS have attracted intense attention including: photocatalytic water splitting [24–25], light-driven CO_2_ reduction [26] and photo-coenzyme regeneration [27] (see Figure 2), which are promising solutions to the energy crisis, greenhouse effect and food shortage, respectively [24,26,28–45].

Photocatalytic water splitting aims to convert water into hydrogen and oxygen. Some studies have focused on only the half-reaction for water splitting, while ignoring the other half-reaction for oxygen production. This is often called photocatalytic hydrogen production (or generation) and can be regarded as a low-configured version of photocatalytic water splitting. As a renewable and nontoxic gas, hydrogen works not only as a clean fuel but also as a feedstock for important chemical production, such as ammonia and methanol. Similarly, light-driven CO_2_ reduction has great potential as a clean fuel supplier, especially for the production of methanol or methane [45–46]. Additionally, with the consumption of CO_2_, APS possibly provides a solution to the greenhouse effect and global warming. Unlike human beings, plants have no need to use CO_2_ as a clean fuel or for to reduce the greenhouse effect. They simply “consume” CO_2_ to produce carbohydrates (e.g., sugar, cellulose). The global demand for food is increasing dramatically with the continuous growth of the world population [47]. An increase in population requires more land, water and energy, which in turn decreases the ability to produce food. Meanwhile, the requirement for more land results in the destruction of an increased area of forests, further disrupting the climate change and the greenhouse effect. Thus, in a global perspective, the energy crisis, climate change, greenhouse effect, air pollution and food shortage are interconnected. Food production in the form of crops relies on the famous Calvin cycle, in which the coenzymes – nicotinamide adenine dinucleotide hydride (NADH) and nicotinamide adenine dinucleotide phosphate hydride (NADPH) – play an important role since they work as the reducing power for the biosynthetic reactions. Despite its advantages, NPS still has a very low energy conversion efficiency (typically <1%) to capture sunlight and CO_2_ for the production of carbohydrates, even after billions of years of evolution, and it is far from its theoretical limit of 30% [48]. This gives great room to develop an improved scientific solution to produce basic food materials with high energy efficiency while circumventing all the problems.

Optofluidic technology has recently exploded with new concepts to enable fine control of light and liquids in microscale structures [39–41]. In fact, NPS is naturally an optofluidic system as it is carried out in microscale organisms (chloroplasts) filled with fluids (see Figure 1), inferring the feasibility to be mimicked by man-made optofluidic structures. Optofluidics can promote the reaction efficiencies due to its advantages such as large surface-area-to-volume ratio [40], fast reaction rate, high-precision manipulation [38], easy flow control and improved mass and photon transfer in the reaction system [49–53]. The small volume of optofluidic systems reduces the diffusion time, dramatically increases the reaction rate on the photocatalyst surface, and reduces the consumption of expensive photocatalysts and enzymes. Therefore, this platform is also useful for the rapid screening of various photocatalysts [54–59]. Inexpensive, parallel tests are beneficial for the rare or expensive chemical reactions, too [60–61]. Moreover, for enzymatic reactions, the flow-based reaction has the great advantage of avoiding product inhibition and cross-reaction [42]. Furthermore, cascaded reactions can be divided into different areas where each region can be set with their own optimal reaction conditions such as temperature, pH and concentration. The various photocatalytic nanomaterials [62] can be designed in different forms in the microreactor inside the optofluidic device, such as in the form of immobilized nanoparticles, films, plugs, droplets, etc.

Fortunately, many works have contributed to the field of APS. The research efforts have mostly focus on aspects such as development of novel catalytic materials, modification of the existed catalytic materials (e.g., noble-gas doping, co-catalyst impregnation, noble-metal loading, plasmonic sensitization and Z-scheme systems), and optofluidics (or microfluidics) based APS.

It should be noted that many efforts have been made to develop bio-photoreactors to culture microorganisms to produce microalgae, bioenergy and biomass [63]. Broadly speaking, these bio-photoreactors also belong to optofluidics-based APS, but they are not covered in this article since many of them use large reactors and are thus not related to microstructures.

The following review will start with a brief introduction on the mechanisms of photocatalysis-based APS (water splitting, CO_2_ reduction and coenzyme regeneration). Then we will introduce the representative designs of these three areas with an emphasis on how they help solve the existing problems in their respective area. Finally, we conclude with a discussion of the technical barriers that hinder their practical application.

### Basic mechanisms of artificial photosynthesis

The APS reaction is not a spontaneous reaction, for example, the Gibbs free energy in water splitting increases by 237 kJ·mol^−1^, and the required energy could be offered by external light. In principle, a semiconductor photocatalyst (e.g., TiO_2_, C_3_N_4_) absorbs the appropriate photon (*h*ν *≥ E*_0_, where *E*_0_ is the bandgap of the semiconductor photocatalyst) to excite an electron in the conduction band, leaving a hole in the valence band. The electron moves to the surface-active sites for surface redox reactions [64]. The activation equations for water splitting, CO_2_ photoreduction and coenzyme regeneration are as follows:

[1]
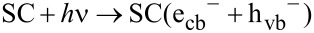


[2]
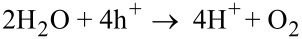


[3]
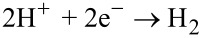


[4]
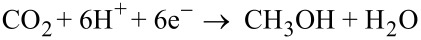


[5]
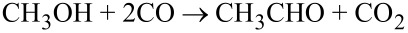


[6]
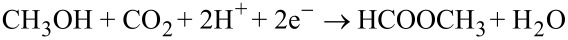


[7]
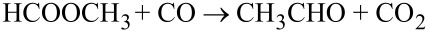


[8]
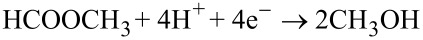


[9]
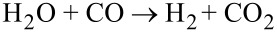


[10]
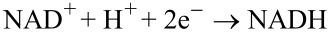


Equation 1 represents the formation of the electron–hole pair. Equation 2 and Equation 3 describe the water splitting process. Equations 4–9 present the CO_2_ reduction processes and Equation 10 is the typical NAD^+^ regeneration. For clearly uncovering the chemical mechanism from the reactants to products, some intermediate processes are reasonably ignored.

All these three items (i.e., water splitting, CO_2_ reduction and coenzyme regeneration) utilize the electrons on the reduction site, as shown in Figure 2. However, recombination of photoexcited electrons and holes may occur. Even after the electrons are moved to the surface of photocatalysts, some of them would be wasted due to recombination if the electrons are not used immediately for the redox reactions [65–67].

**Figure 3:**
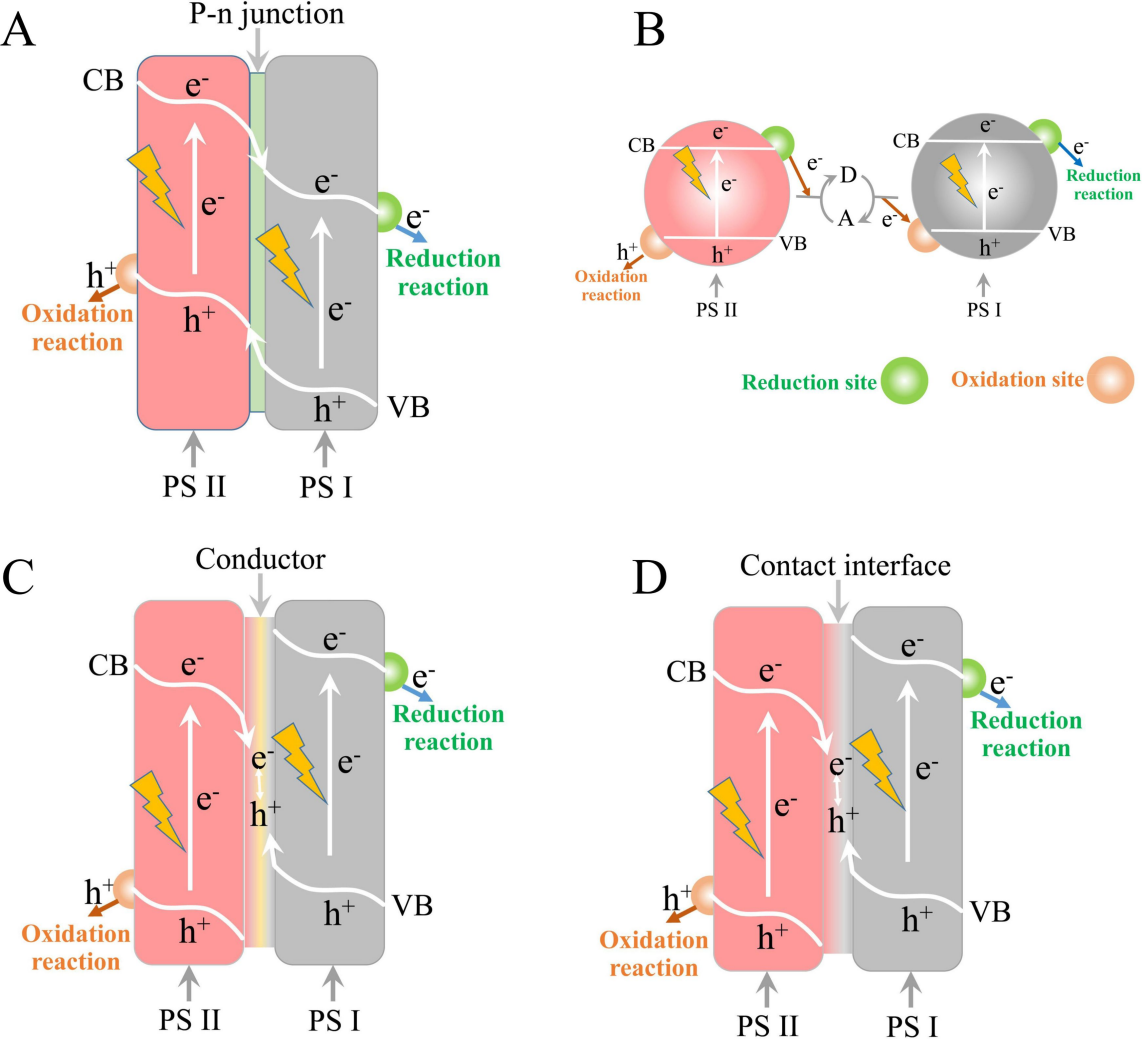
Schematic diagrams of heterojunction and Z-scheme systems. Transfer of charge carriers in (A) the heterojunction-type photocatalytic system, (B) the Z-scheme PS-A/D-PS system, (C) the Z-scheme PS-C-PS system, and (D) the Z-scheme electron PS-PS system. Adapted from [68], copyright 2014 Wiley-VCH Verlag GmbH & Co. KGaA. PS stands for photosystem.

The desirable features of a photocatalyst include wide-range absorption, long-term stability, fast electron–hole separation, and strong redox powers. However, it is difficult to have all of these features in a single photocatalyst. Thereby, a simple heterojunction of two or more photocatalysts and artificial Z-scheme photocatalytic systems have been developed [68]. Figure 3A shows the charge carrier transfer in a heterojunction-type photocatalytic system, in which the photo-generated electrons and holes are separated in space to suppress the undesirable recombination. However, when the charge carriers are transferred to lower potentials, the redox ability of these electrons and holes is weakened. Then, another type of photocatalytic system is explored, as shown in Figure 3B. The electron acceptor/donor (A/D) pair is introduced to form the Z-scheme system, known as the PS-A/D-PS system. Since the electron acceptor (A) can react with both the photogenerated electron in PS I and PS II, the electron donor (D) can react with both the photogenerated hole in PS I and PS II, and backward reactions would occur, leading to a significant waste of photogenerated electrons and holes. In another design, the A/D pair is replaced by the conductor (C) to form the PS-C-PS system, as shown in Figure 3C. The inserted conductor acts as the electron mediator and forms the ohmic contact with low contact resistance between PS II and PS I. Through the ohmic contact, the photogenerated electrons from PS II directly recombine with the photogenerated holes from PS I, reducing the electron transfer distance and avoiding the backward reaction in the PS-A/D-PS system. Another simpler design using the solid–solid contact (PS-PS system) is illustrated in Figure 3D. On the contact interface, many defects are easily aggregated, causing the energy levels to be quasi-continuous for the ohmic contact. Besides, the biomimetic or bioinspired strategy showed the most interesting results. Zhou et al. reported a light-harvesting antenna-network inspired polymeric semiconductor-based hybrid nano-system in which water and CO_2_ were catalyzed to form H_2_ and CO in this integrated system [66]. Jiang et al. showed a thylakoid-inspired multishell g-C_3_N_4_ nanocapsule with orderly stacked nanostructures, which exhibited enhanced visible-light harvesting and electron-transfer properties for high-efficiency photocatalysis [67].

In summary, the basic mechanism of APS is comprised of three processes: (1) generation of charge carriers (i.e., electrons and holes), (2) separation and transfer of charge carriers, and (3) chemical reactions between surface species and charge carriers. Based on this mechanism, various materials have been developed to improve the photocatalytic efficiency [69–72]. Furthermore, by taking advantage of the properties of optofluidics, many studies with interesting improvements have been reported. We will now review these in terms of water splitting, CO_2_ reduction and coenzyme regeneration.

### Microreactors for artificial photosynthesis

An optofluidic microreactor is a versatile platform for combining new materials and characterizing their reaction kinetics without expensive bulky setups. Recent developments in optofluidics has allowed for the advancement of APS technology by use of the microreactors.

#### Water splitting

**Figure 4:**
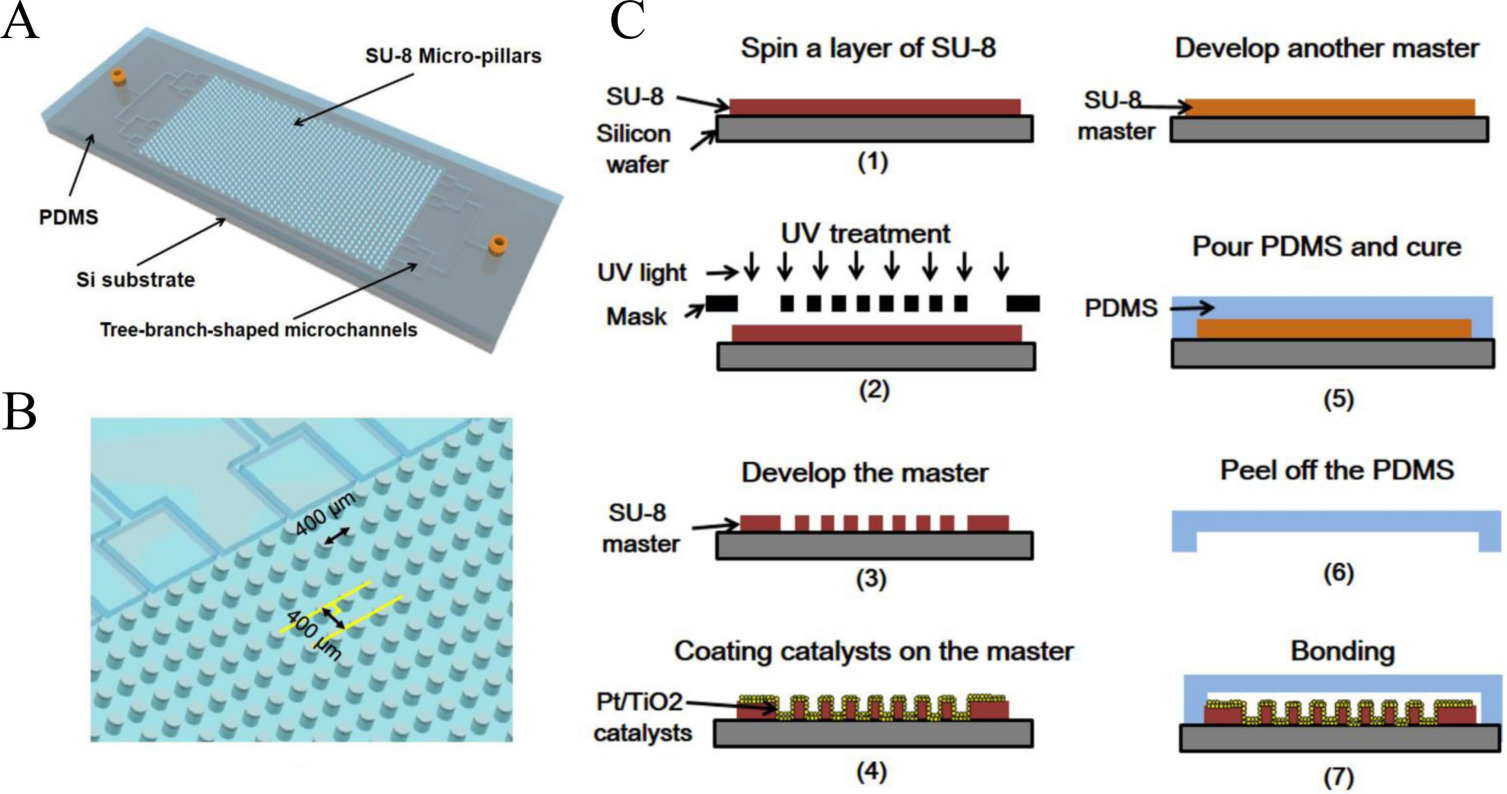
(A) Schematic of the high-surface-area optofluidic microreactor with micropillar structure. (B) Staggered micropillars in the reaction chamber. (C) Fabrication procedure of the optofluidic microreactor with the catalyst-coated micropillars. Reprint with the permission from [74], copyright 2014 Elsevier Ltd.

In the early studies on optofluidic-based water splitting, the optofluidic device often employed sol–gel catalysts on planar channels. For example, Erickson et al. demonstrated a planar setup with TiO_2_–Pt to process the water splitting reaction [73]. The reaction was mediated by I^−^/IO_3_^−^ redox pairs, belonging to the PS-A/D-PS system. After the reaction, the optofluidic device showed ≈2-fold improvement in the reaction rates as compared to the traditional bulk method. Nevertheless, this planar design still showed an unsatisfactory performance of hydrogen generation due to a limited active surface area and low mass transfer rate. In another work, Wang et al. proposed an optofluidic microreactor with staggered micropillars in the reaction microchamber [74], as shown in Figure 4. Such structure has four favorable features: (1) enlarged surface area for loading catalysts; (2) perturbation to the liquid flow for rapid mixing; (3) shortened transfer length and enhanced mass transfer; and (4) increased active surface area. With these advantages, the reaction rate could be increased by 56% as compared to the conventional planar microreactors.

**Figure 5:**
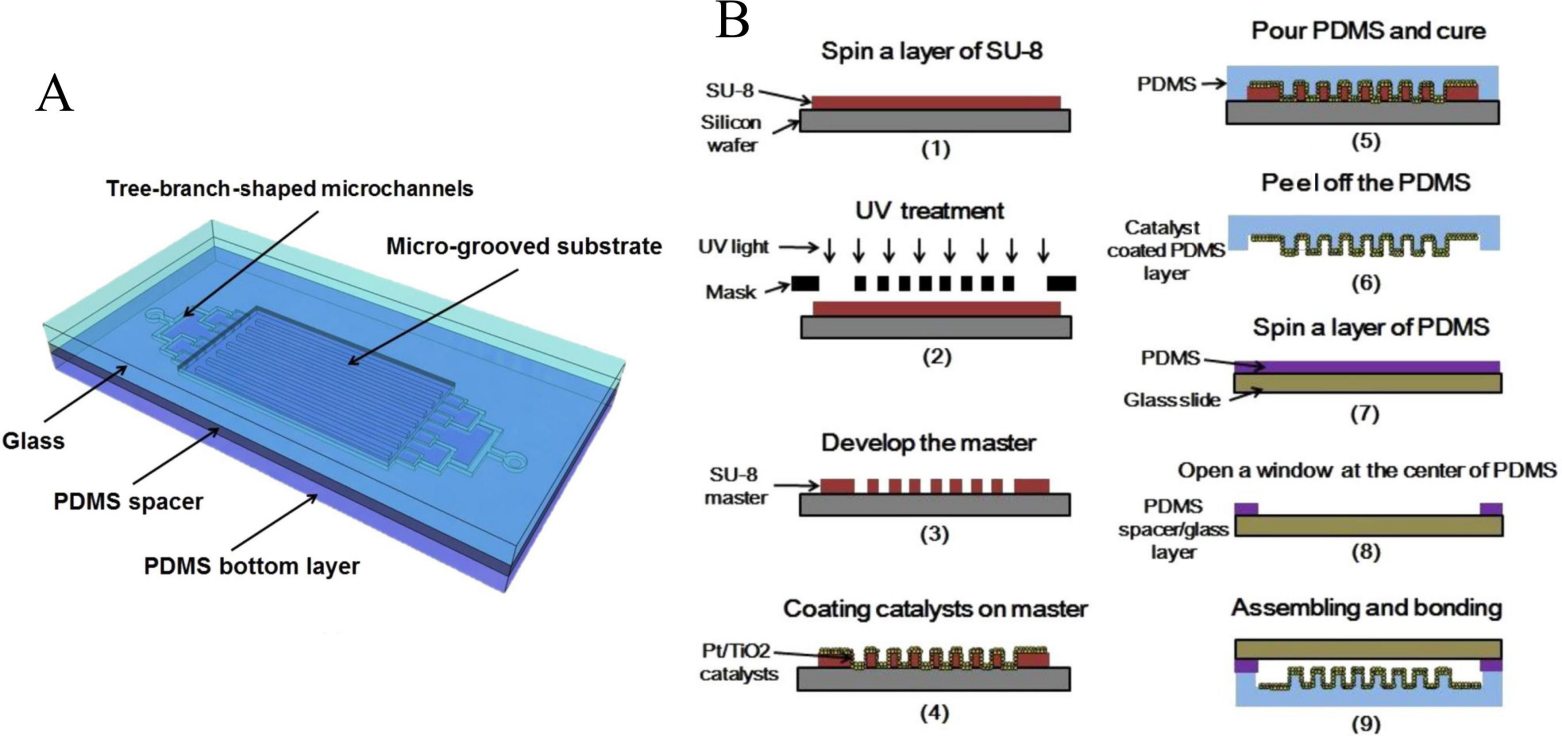
(A) Schematic of the high-surface-area optofluidic microreactor with micro-grooved structure. (B) Fabrication procedure of the optofluidic microreactor with the catalysts on the PDMS substrate. Adapted from [36], copyright 2015 Elsevier Ltd.

However, a new problem emerges: the direct coating methods are unable to load catalysts firmly and uniformly on the PDMS substrate. Zhang et al. proposed a new casting transfer method for loading catalysts on the PDMS substrate [36], as shown in Figure 5. This method exhibited critically higher durability and better hydrogen production rate than the conventional ones.

#### CO_2_ reduction

**Figure 6:**
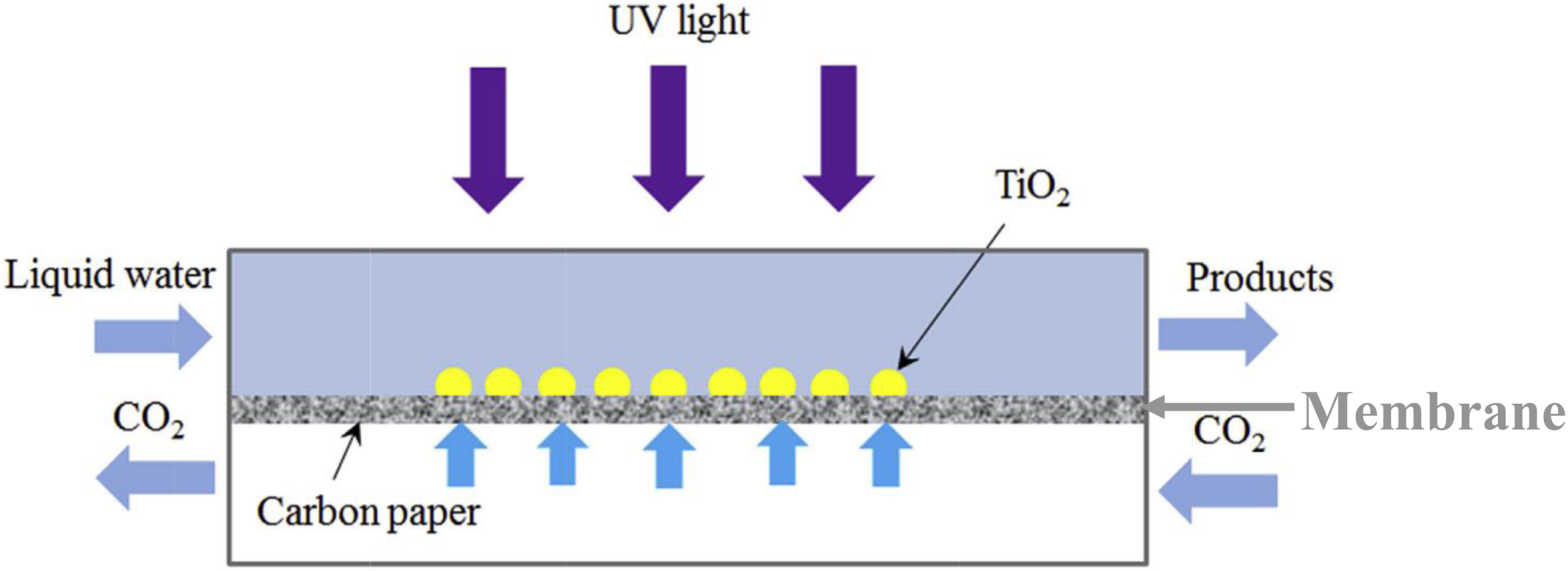
Schematic of the optofluidic membrane microreactor for photocatalytic CO_2_ reduction. Adapted from [76], copyright 2016 Elsevier Ltd.

Optofluidic microreactors have been firstly applied for water purification [50], water splitting [73], photocatalytic fuel cells [75] and then CO_2_ reduction [76]. Chen et al. combined the optofluidics with the TiO_2_/carbon paper composite membrane for the photoreduction of CO_2_ [76], as shown in Figure 6. Using this device, they studied the factors that affected the methanol yield (such as flow rate, light intensity and catalyst loading) and obtained a high reduction result in comparison to the reported data. Other membrane-based reactors were reported as well, for instance, mesoporous CdS/TiO_2_/SBA-15@carbon paper composite membranes [77] and copper-decorated TiO_2_ nanorod thin films [78].

**Figure 7:**
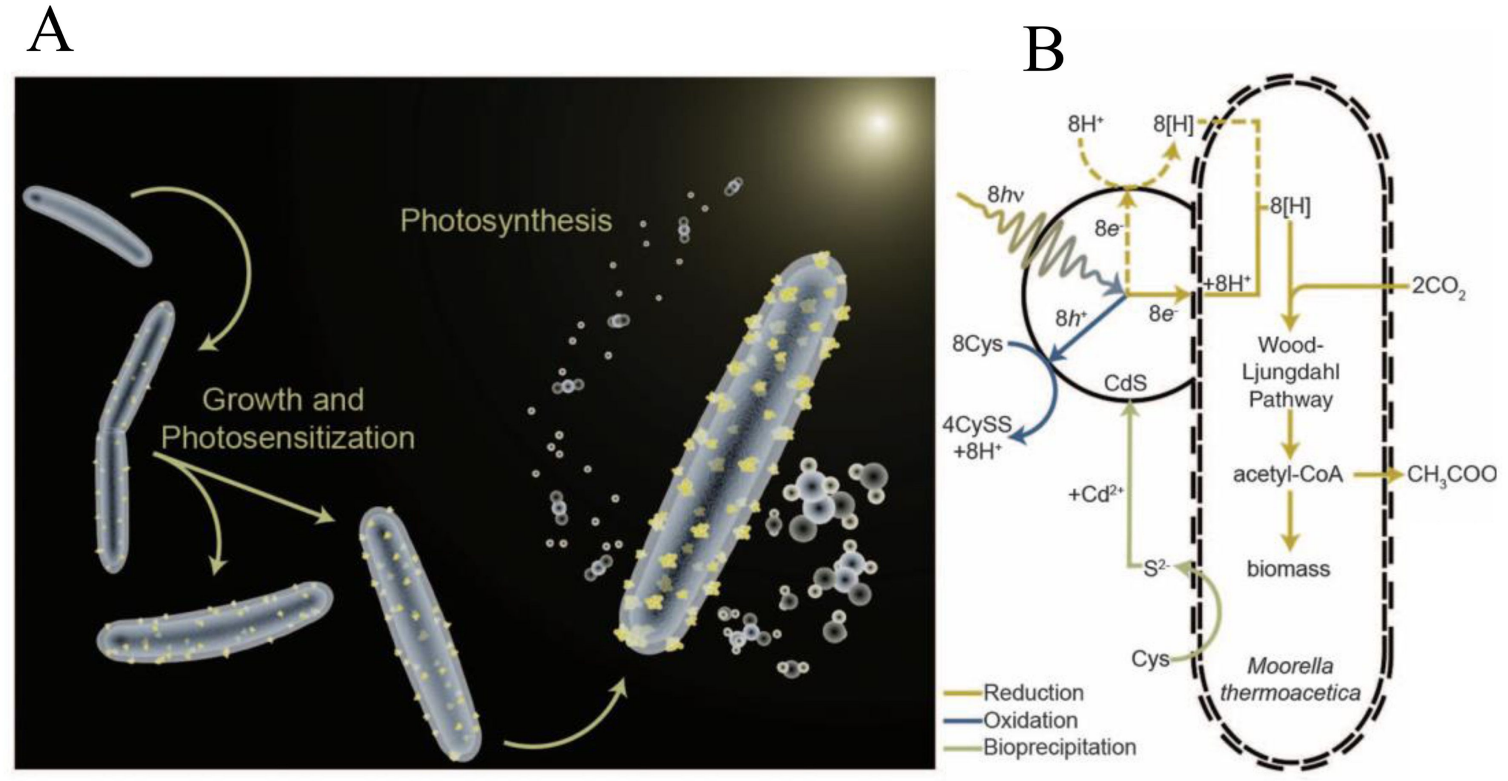
(A) Bacterium–CdS hybrid system that has CdS nanoparticles on the bacterium membrane (yellow particles). (B) Pathway diagram for the light harvesting and the photosynthetic conversion of CO_2_ to acetic acid with the bacterial enzyme system. Adapted from [79], copyright 2016 American Association for the Advancement of Science.

In addition to the PDMS/PMMA-based microchannel reactors, bacterium has been shown to be a good microreactor as well. Yang et al. developed a hybrid approach that combined highly efficient light harvesting of inorganic semiconductors with biocatalysts [79]. As shown in Figure 7, they induced a non-photosynthetic bacterium with biologically precipitated CdS nanoparticles, enabling the photosynthesis of acetic acid from CO_2_. The CdS nanoparticles functioned as the light harvester. This self-augmented biological system selectively produced acetic acid continuously over several days, demonstrating a novel CO_2_ reduction microreactor.

#### Coenzyme regeneration

APS-based coenzyme regeneration has attracted less attention as compared to water splitting and CO_2_ reduction [80], but significant progress had already been made before it was combined with the optofluidics [81]. Park et al. developed CdS quantum-dot-sensitized TiO_2_ nanotube arrays for the photo-regeneration of nicotinamide cofactors [82]. They also used SiO_2_-supported CdS quantum dots for sustainable NADH regeneration [83]. However, the toxicity and photoinduced instability of CdS limited the application of this material. Liu et al. used carbon nitride (C_3_N_4_), a stable and environmental friendly material [84], for NADH regeneration [85–87]. For example, one bioinspired method utilized the diatom as the C_3_N_4_ formation templet, enlarging the specific surface area for enhanced light trapping and scattering and eventually high photocatalytic efficiency [27]. This research is mostly based on the slurry method. Optofluidics-based coenzyme regeneration appeared only in recent years.

**Figure 8:**
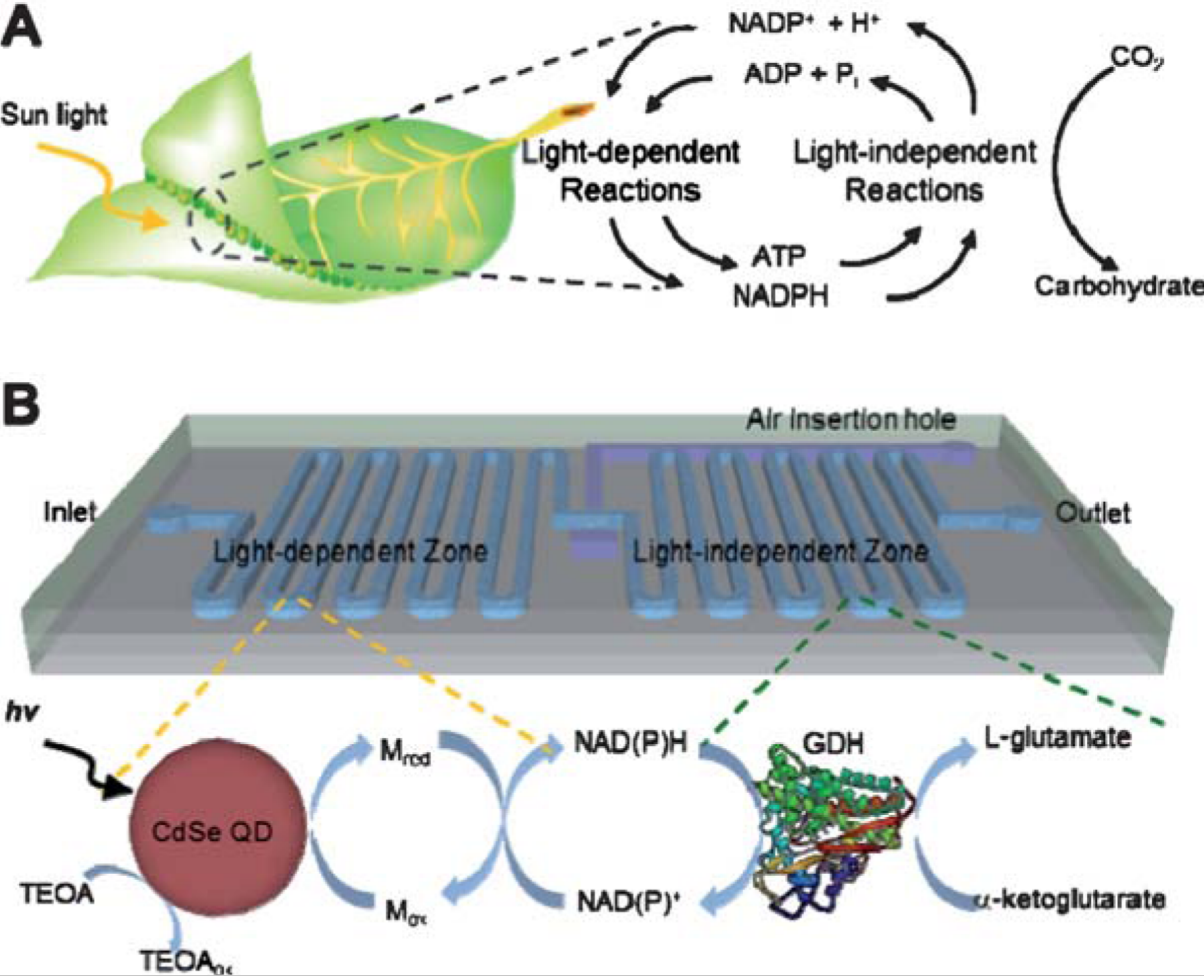
Microfluidic APS platform that incorporates quantum dots and redox enzymes for photoenzymatic synthesis. (A) Concept and (B) besign of microreactor in which the cofactor regeneration takes place in the light-dependent reaction zone and the enzymatic synthesis in the light-independent zone. Adapted from [88], copyright 2011 Royal Society of Chemistry.

**Figure 9:**
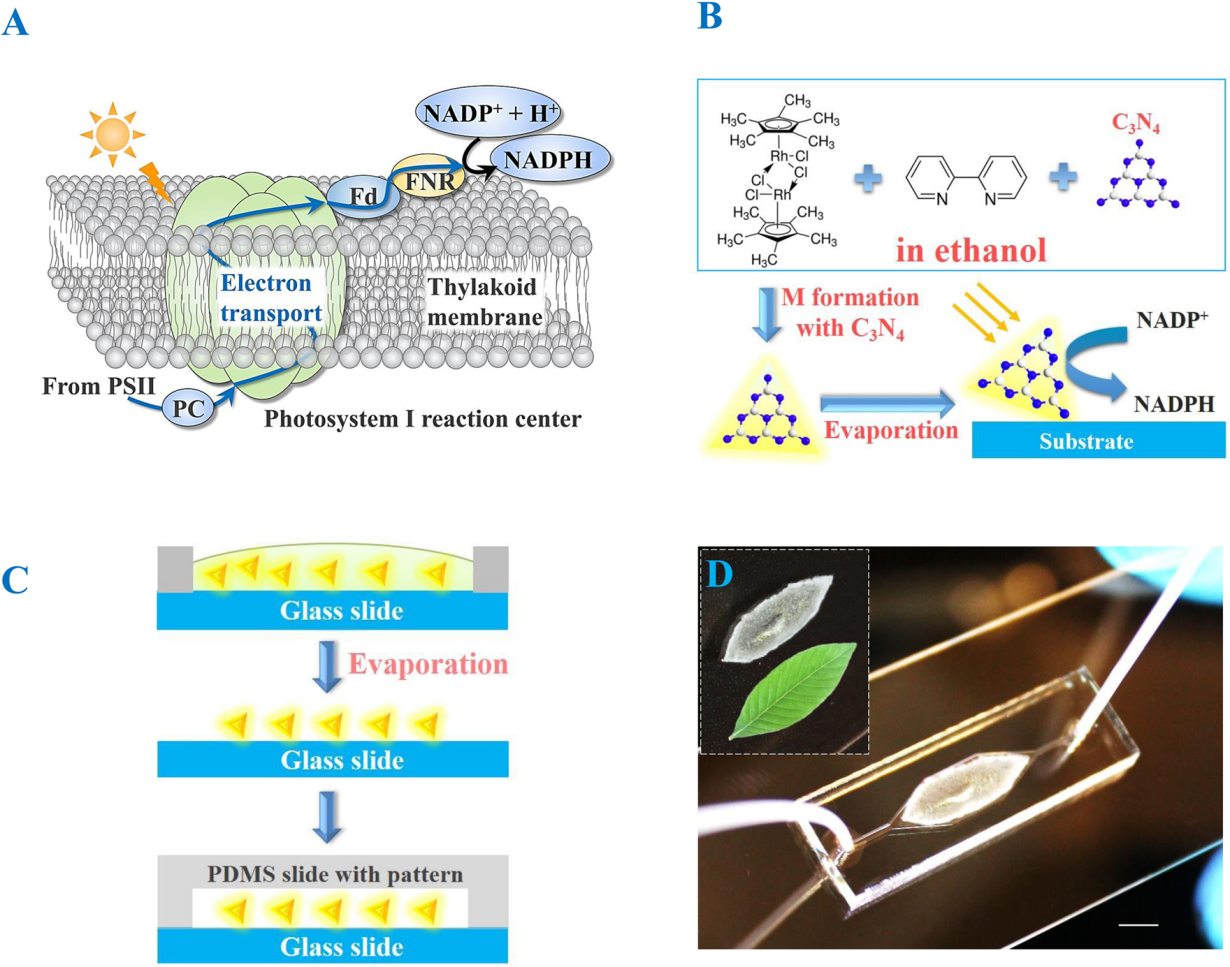
Microfluidic chip based artificial photosystem I. (A) Schematic illustration of the PS I reaction center. (B) One-step fabrication process of the immobilized artificial PS I (IAPSI) in the form of the g-C_3_N_4_-M film. (C) Simple procedures to fabricate the IAPSI microreactor. (D) Photograph of the as-fabricated IAPSI microreactor, in which the inset presents the leaf-like shape of g-C_3_N_4_-M. The scale bar is 2 mm. Adapted from [89], copyright 2016 The Royal Society of Chemistry.

Park et al. presented an optofluidic device that incorporated quantum dots and redox enzymes for photo-enzymatic synthesis [88]. As shown in Figure 8, the microchannel was separated into two parts by a valve, the light-dependent reaction zone for NADH regeneration in the upstream of microchannel and the light-independent zone for enzymatic synthesis in the downstream. Our group reported a optofluidic chip-based artificial PS I using a novel one-step fabrication method (see Figure 9), which outperformed the traditional methods in several aspects in terms of facile synthesis, promotion of the combination of g-C_3_N_4_ and electron mediator through π–π stacking, in addition to a significantly enhanced coenzyme regeneration rate [89]. Coenzyme regeneration is also of great importance in CO_2_ reduction with the help of enzymes. Formaldehyde dehydrogenase is a typical one that selectively achieves methanol formation from CO_2_ with the depletion of NADH to NAD^+^ [46]. The continuous regeneration of NADH enables the continuous reduction of CO_2_.

## Conclusion

APS is a promising way to utilize solar energy and a prospective solution for the energy crisis, greenhouse effect and coenzyme utilization. An effective photocatalyst is expected to have wide-range absorption, long-term stability, fast electron–hole separation, and strong redox power. The heterojunction and Z-scheme systems are the important most research results to date. Based on these systems, various materials have been developed for the improvement of photocatalytic efficiency [90–94]. Besides, biomimetic or bioinspired strategies for the synthesis of semiconductor materials represents a significant advancement in the development of high-efficiency and cost-effective visible-light photocatalysts for solar energy conversion [65–67]. Given the advantages of optofluidic systems, more studies with significant improvement are expected.

The optofluidic planar microreactor has shown to be a versatile platform for superior performance in water splitting, CO_2_ reduction and coenzyme regeneration. However, there are still some problems. One problem is the limited production amount caused by the small volume of the microreactor. For high throughput and adequate production, a feasible solution may involve the combination of two approaches: to parallelize the microreactors to form an array and to enlarge the microchannel in the lateral direction into a planar chamber [50,95–98]. Another problem is the lack of an integrated on-chip detection method. After the reaction, the production products should be collected and then analyzed by the bulky, expensive, traditional off-chip equipment, such as a UV–vis spectrometer or high-performance liquid chromatography and gas chromatography mass spectrometer. As demonstrated by our group in a work on microfluidic water purification [99], on-chip detection would make it convenient to monitor the reaction process in real time, to probe the intermediate reactions/productions and to study the reaction kinetics.

In view of the many merits induced by optofluidics, the microreactors may be also introduced to other APS systems such as nitrogen fixation (e.g., NH_4_) [100], CH_4_ [101–106], CO [107–111], formaldehyde [112–113], methanol [114–116], and formic acid [117]. More profound and meaningful work is expected to appear in the field of optofluidics-based APS since the optofluidic devices are versatile and can be integrated together with other functions, for instance, deoxygenation, temperature control, electricity/magnetic field and pressure [118–121].
